# The Most Widespread Symbiosis on Earth

**DOI:** 10.1371/journal.pbio.0040239

**Published:** 2006-07-11

**Authors:** Andreas Brachmann, Martin Parniske

The interaction between plant roots and Glomeromycota fungi [ 1]—arbuscular mycorrhiza (AM)—is probably the most important symbiotic association in nature. More than 80% of all higher land plant species are able to benefit from the fungus' ability to extract nutrients, mostly phosphorus, from the soil. In exchange, it has been estimated that worldwide up to 20% of all photosynthetically fixed carbon might be delivered to the fungal partner. This makes it the most widespread symbiosis on earth [ 2–4]. Yet, despite its ecological importance, astonishingly little is known about the molecular mechanisms involved in this contact. In this issue of *PLoS Biology*, Besserer et al. [ 5] study how plant roots are recognized by their fungal partner and demonstrate that these events generalize across species.

Fossil evidence shows AM symbiosis to be at least as old as the earliest land plants [ 6, 7]. It has been suggested that colonization of the land surface was dependent on the fungal symbiont's ability to forage into the soil for inorganic nutrients and water. This interaction apparently proved to be so successful that even after development of functional plant root structures, it was still retained in most plant families. However, a surprising promiscuity is observed in this symbiosis since, at least under laboratory conditions, many plant–fungus combinations lead to symbiotic structures. This lack of specificity is rather puzzling, considering the very long period of coevolution and the complete lack of sexual reproduction within the fungal partner, and points to a common mechanism of recognition.

Two important discoveries demonstrated that the early interaction between plant root and fungus involves mutual release of diffusible signaling molecules. Plant roots release “branching factors” (BFs) that induce morphological and cytological responses in the approaching fungal hyphae [ 8]. Among these, hyphal branching is the most prominent morphological change that can be attributed to fungus–plant recognition. In turn, fungal hyphae produce “myc factors” that lead to the transcriptional induction of symbiosis-related genes in the host root [ 9]. These pre-symbiotic recognition events are pre-requisite for formation of fungal appressoria structures on the root surface, invasion and colonization of fungal hyphae inside the root cortex, and, finally, the formation of highly branched, tree-like fungal structures (arbuscules) inside plant cells. The surface increase associated with arbuscule formation is believed to aid nutrient exchange between the partners.

Recently, Akiyama et al. [ 10] provided a major breakthrough in our understanding of the very early recognition events in this process. They identified strigolactones as the BFs released by the AM plant Lotus japonicus that trigger hyphal branching in the AM fungus Gigaspora margarita. Strigolactones belong to the sesquiterpene lactones, which are believed to have a wide distribution in the plant kingdom [ 11]. Curiously, the same class of molecules was described exactly 40 years ago as a germination stimulant for seeds of the parasitic weeds *Striga* (witchweed) and Orobranche that attack plant roots and deprive them of water and nutrients [ 12]. Strigolactones occur with different substitutions, yet very little specificity was observed when different structures of natural and synthetic origin were tested for their activity on Gi. margarita. The widespread occurrence of strigolactones and this low structural specificity may underlie the promiscuity of AM fungi.

Still, open questions remained. The Glomeromycota are considered the fifth fungal phylum [ 1] and their common ancestor dates back 600 million years [ 7], yet all of these fungi exist in symbiosis with phototrophic organisms. Members of this phylum are more diverged from each other than the evolutionary younger basidiomycetes or ascomycetes, which have conquered very different niches during their evolution. Is responsiveness to strigolactones a general phenomenon within this phylum despite its old age? Besserer et al. [ 5] could show responses to strigolactones and synthetic derivatives, but not other sesquiterpene lactones, in three representatives of phylogenetically diverged groups of AM fungi: Gi. rosea, Glomus intraradices, and Gl. claroideum, thus providing important confirmation of a general recognition mechanism. This widespread strigolactone perception system suggests that already the earliest land plants used this class of molecules to communicate with their symbiotic partners.

Interestingly, the induced morphological changes seem to be dependent on the fungal species. Whereas in both Gi. margarita and Gi. rosea spore germination occurs by itself and strigolactones produce an increase in hyphal branching, the main strigolactone effect on Gl. intraradices and Gl. claroideum is an elevated germination rate.

Moreover, Besserer et al. [ 5] were able to demonstrate that application of strigolactone to these fungi led to not only morphological changes but also a rapid increase in mitochondrial density and respiration, both hallmarks of pre-symbiotic reprogramming of the fungus [ 8, 13]. Perception of the plant signal seems to activate the breakdown of storage lipids, which enables the fungus to forage and ramify in order to enhance the chance of an encounter with plant roots. The high instability of strigolactones fits well with this role as a short-range signaling factor.

The finding that plant secondary compounds can modulate fungal development invites speculations about possible additional roles for strigolactones during symbiosis. For example, it is completely unclear what signal triggers the profuse hyphal branching observed during arbuscule development. Strigolactones are obvious candidates to induce such developmental changes in the fungus. What is still missing is a quantitative assessment of the importance of strigolactones for symbiosis. An important tool would be plants unable to produce strigolactones. Such genetic strategies have been very successful in eliminating a major role for flavonoids during AM development [ 11].

The ability to activate the pre-symbiotic stage of the fungus in a synchronized fashion will certainly help to unravel associated molecular events. Production and secretion of fungal symbiotic signaling factor(s) appears to be dependent on perception of the plant host [ 9] and may be induced after application of strigolactones. Thus, molecular identification of the BF and demonstration of biological activity of synthetic derivatives opens up interesting possibilities for future research on the AM fungus.

## Abbreviations

AM: arbuscular mycorrhiza
BF: branching factor
